# Coherent destruction of tunneling in two-level system driven across avoided crossing via photon statistics

**DOI:** 10.1038/srep28959

**Published:** 2016-06-29

**Authors:** Qiang Miao, Yujun Zheng

**Affiliations:** 1 School of Physics, Shandong University, Jinan 250100, China

## Abstract

In this paper, the nature of the multi-order resonance and coherent destruction of tunneling (CDT) for two-level system driven cross avoided crossing is investigated by employing the emitted photons 〈*N*〉 and the Mandel’s *Q* parameter based on the photon counting statistics. An asymmetric feature of CDT is shown in the spectrum of Mandel’s *Q* parameter. Also, the CDT can be employed to suppress the spontaneous decay and prolong waiting time noticeably. The photon emission pattern is of monotonicity in strong relaxation, and homogeneity in pure dephasing regime, respectively.

Two-level system (TLS) driven across avoided crossing is pervasive in physical problems. Despite its simplicity, it is an important subject to investigate various fundamental phenomena and lay a rich basis for real-world technologies. Recently, TLS has obtained plenty of attention in quantum information processing (QIP) as well as fundamental studies for the single quantum system, such as superconductor qubit^1^,^2^,^3^,^4^,^5^,^6^, single spin or charge in quantum dots^7^,^8^,^9^,^10^ and trapped ion^11^. This progress provides an elementary building block in QIP and an ideal testing platform for probing single quantum phenomena.

For certain driving parameters, the TLS undergoes coherent destruction of tunneling (CDT) where the quantum tunneling is destroyed^12^,^13^,^14^. The initial quantum state is frozen, which happens at the crossing point of isolated quasi-energies levels. Besides, the strong driving can also lead to the multi-order transition at certain resonance condition. All these phenomena could be understood by Landau-Zener-Stückelberg (LZS) interference^8^,^9^,^10^,^15^,^16^,^17^,^18^,^19^ around the avoided crossing point, in which sequential constructive or destructive interferences dominate the dynamical evolution. To analyze this interference pattern, several theoretical approaches have been developed, such as rotating-wave approximation (RWA) method^1^,^19^, Floquet formulation combining generalized Van Vleck (GVV) approximation method^20^,^21^, etc. The former method makes contributions to simplifying the TLS problem, and the latter sheds light on the understanding of the ac Stark level shift and CDT shift, etc.

In the realistic physical systems, the TLS has to be considered as a dissipative system. A bath-TLS coupling model has been investigated in refs 22, 23, 24, 25 comprehensively, in which the dissipative rates are parameter-dependent. Alternatively, the optical TLS with phenomenological dissipation constants also shows its charm, e.g., a geometric phase-shift gate has been proposed based on the CDT effect in this case recently^26^. In the optical TLS, it could emit photons via inevitable spontaneous radiation. For each photon emission event, the TLS loses its memory and is reset back to ground state eventually. By monitoring the photon emission events, one can obtain more dynamical information of system, especially, as a secondary order moment, the Mandel’s *Q* parameter are much sensitive to the dynamics. Besides, the time waiting for spontaneous decay can be used to measure the ability of preserving memory, which is crucial in the quantum control.

By employing the photon counting statistics of generating function technique^27^,^28^,^29^,^30^, in this paper, we investigate the representation of CDT via photon emission from TLS, and test the new physics beyond the approximation methods mentioned above. We present the ac Stark shift and CDT shift in weak and moderate driving field where RWA approximation fails. We find the Mandel’s *Q* parameter is sensitive to detuning nearby CDT position and shows an asymmetric spectrum beyond GVV approximation. We show CDT can be employed to suppress photon emission, but it is also sensitive to disturbance from environment. The new nature of the destruction of CDT by strong dissipation, the monotonicity or homogeneity pattern of photon emission, is presented using the emitted photons from the TLS.

This paper is organized as follows. Initially, we introduce the TLS Hamiltonian and its expressions under RWA and GVV approximations. Also, we review the theoretical framework of photon counting statistics by employing the generating function. Subsequently, we show the numerical results in different regimes: weak relaxation regime under moderate and fast driving; strong relaxation or pure dephasing regime under fast driving. The conclusion is given in the end.

## Theoretical Framework

### Model system and Hamiltonian

#### General considerations

We consider a two-level system (TLS) described by the Hamiltonian
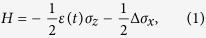
where *ε*(*t*) is the time-dependent tunable control parameter, Δ is the coupling strength between the ground state |*g*〉 and excited state |*e*〉. *σ*_*x*_ and *σ*_*z*_ are the Pauli matrices and we set *ħ* = 1 throughout this paper. The TLS Hamiltonian Eq. 1 has been employed to investigate various interesting questions^1^,^2^,^8^,^9^,^10^,^19^,^20^,^21^,^26^,^31^

In this paper, we consider the case that *ε*(*t*) is modulated by
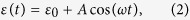
where *A* is driving amplitude, *ω* denotes driving frequency and *ε*_0_ represents static detuning, respectively.

The TLS Hamiltonian Eq. 1 can, after considering Eq. 2, also be separated into two parts: time-independent component *H*_0_ and time-dependent component *V*(*t*), namely
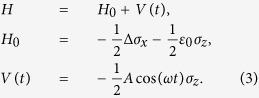


In the expression of Eq. 3, *H*_0_ is static configuration of TLS, and *V*(*t*) provides crucial driving parameters.

#### Hamiltonian in RWA form

Following refs 1,19,31, we take the TLS Hamiltonian described by Eq. 3 in a rotating frame, making a transformation from laboratory frame by rotating operator *W*(*t*). The rotating operator *W*(*t*) is defined as follows
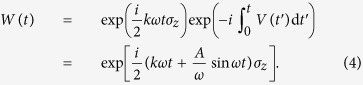


The wave function in the rotating frame |*ψ*′〉 is given by
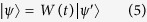
and the Schrödinger equation in rotating frame is
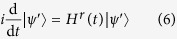
with
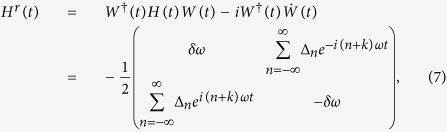
where *δω* = *ε*_0_ − *kω*, 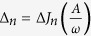, and *J*_*n*_(·) is the Bessel function of the first kind.

Under the condition of near *k*-th resonance (namely, *δω* ~ 0), and in fast driving regime 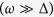, Eq. 7 can be approximately expressed as follows
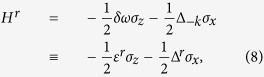
where *ε*^*r*^ = *δω* and Δ^*r*^ = Δ_−*k*_.

In this approximation, the Hamiltonian of Eq. 8 is valid for strong driving amplitude *A*^20^,^21^,^31^.

#### Hamiltonian in Floquet and GVV perturbation theory

A system driven periodically could be treated by Floquet formalism, where a time-dependent problem can be transformed into an equivalent time-independent problem in the infinite dimensional Floquet states space. In the Floquet space, the quasi-energy of the infinite dimensional Hamiltonian is derived, and the resonance transition would occur nearby avoided crossing position of quasi-energy levels^32^,^33^. Also, the Van Vleck perturbation theory allows us to focus on near-degenerate state space and neglect other perturbative manifolds. By a unitary transformation up to a certain order perturbation, one can project the infinite Hamiltonian onto a simple effective one^34^. Here, the TLS Hamiltonian can be expanded by employing Floquet bases of |*g*, *n*〉 and |*e*, *n*〉 with quasi-energies 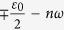, and the effective Hamiltonian for *k*-th resonance up to the third-order perturbation Δ/2 is given by^20^,^21^
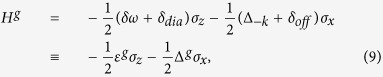
where *ε*^*g*^ = *δω* + *δ*_*dia*_ and Δ^*g*^ = Δ_−*k*_ + *δ*_*off*_, with
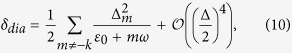
and
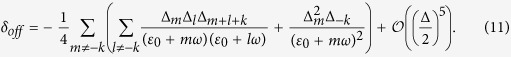


Clearly, *H*^*g*^ for the first-order of Δ/2 recovers the expression of RWA approximation. And the second-order amendment *δ*_*dia*_ represents the ac Stark level shift in diagonal term, leading to a shift of *k*-th resonance peak. In moderate or weak driving regime, namely *ω* ~ Δ or *A* ~ *ω*, the *δ*_*dia*_ is dominant. As for the third-order amendment *δ*_*off*_ in off-diagonal term, it causes a CDT shift relative to the node of Bessel function *J*_*n*_(*A*/*ω*). While in fast regime 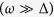, *δ*_*off*_ and *δ*_*dia*_ can be neglected.

### Dynamics and Generating functions

In the dissipative case, the quantum system can be considered in Loiuville space via density matrix. The time evolution of density matrix *ρ*(*t*) of quantum system obeys the Liouville-von Neumann equation,
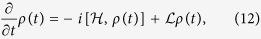
where 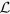 is the superoperator including the spontaneous emission rate Γ and pure dephasing rate *k*_*p*_. Adopting general TLS Hamiltonian, the equations of density matrix Eq. 12 can also be written as follows^34^
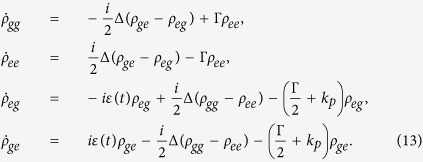


To present the dynamical behaviors of the CDT for TLS and corresponding properties via emitted photons, in this paper, we employ the theoretical framework of photon counting statistics by generating function. This approach has been employed to investigate the single-molecule stochastic kinetics; the control of few photons emission etc.^27^,^28^,^29^,^30^,^35^,^36^,^37^,^38^,^39^. The generating function of photon emission is defined by^27^,^28^,^29^
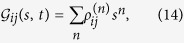
where 
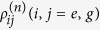
 represents the portion of density matrix corresponding to that *n* photons have been spontaneously emitted. *s* is an auxiliary parameter counting the photon emission events.

The generating equations can, by employing Eqs 13 and 14, be written as follows^27^,^28^,^29^,
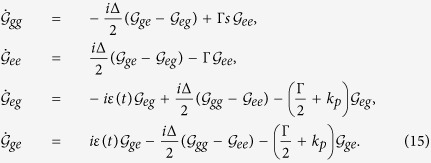


By employing Bloch vector notation^27^,^28^,^29^,
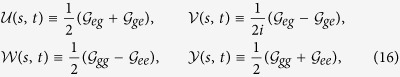


Eq. 15 can also be written as
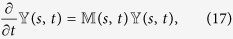
where 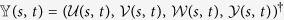 and 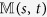 are given by
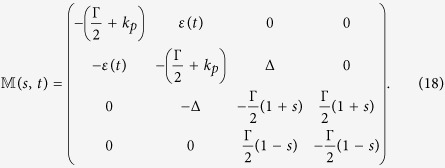


The statistical properties of photon emission can be extracted from 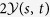 as follows:
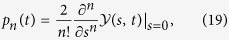

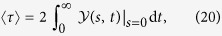

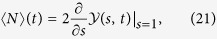

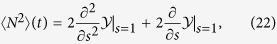
where *p*_*n*_ is the probability of *n* photons have been emitted in time interval [0, *t*], 〈*τ*〉 is average waiting time, 〈*N*〉 (*t*) is first moment, namely average photon emission numbers and 〈*N*^2^〉 (*t*) is secondary moment. And, the Mandel’s *Q* parameter
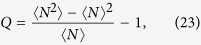
follows immediately, which characterizes the statistical properties of emitted photons. The case of *Q* < 0 is called sub-Poissonian distribution (anti-bunching behavior) and *Q* > 0 is named super-Poissonian distribution (bunching behavior).

It is worth noting that we can employ the different expressions of Hamiltonian, such as Eq. 1 (exact), Eq. 8 (RWA approximation) and Eq. 9 (GVV approximation) to show their different dynamical behaviors via emitted photons.

## Numerical Results and Discussions

Based on the theoretical framework above, in this section, we explore the different dynamical properties of the resonance and CDT influences in weak relaxation regime by the three expressions of Eqs 1, 8 and 9 for the TLS system. The destruction of CDT by dissipative effect is also explored.

In our numerical calculations, we use scaled parameters, namely Δ*t*→*t*, *ω*/Δ→*ω*, Γ/Δ→Γ, and *ε*_0_/Δ→*ε*_0_.

### CDT effect via photon emission

In this subsection, we focus on the CDT effect and the multi-order resonance in the weak relaxation 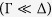. In the weak relaxation, the dynamics of system is dominated by Δ and driving parameters.

#### Moderate driving (*ω* ~ Δ)

##### *δ*_*dia*_ amendment in weak driving

**Figure 1:**
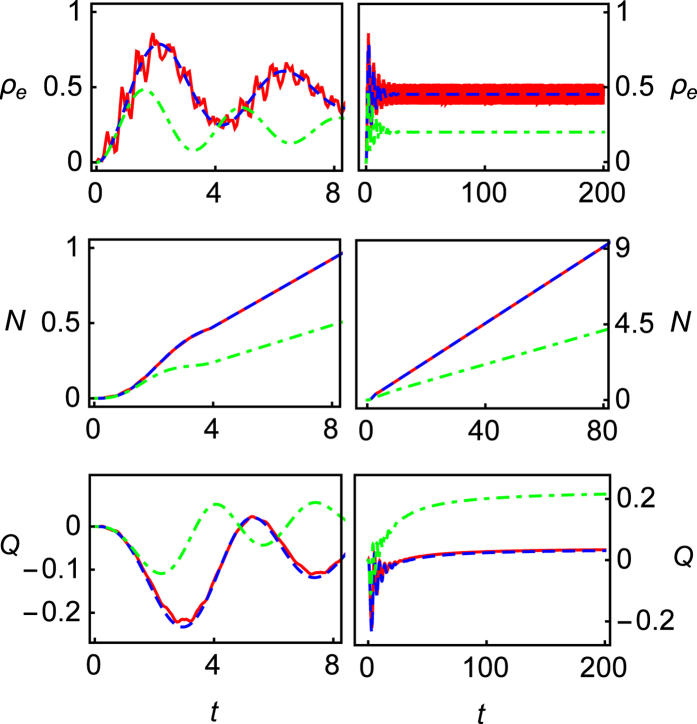
Population *ρ*_*e*_, photon emission 〈*N*〉 and the Mandel’s *Q* parameter as a function of time *t*. The parameters are *ω* = 2, *A*/*ω* = 1.5, *ε*_0_/*ω* = 1.9, Γ = 0.04, *k*_*p*_ = 0. The red lines represent exact numerical results, green dash-dotted lines are the results of RWA approximation, and the blue dashed lines are the results of the second-order GVV approximation. The statistical behaviors of system for short time scale are shown in the left column and those for long time limit are shown in the right column, respectively.

Figure 1 shows the population of excited state *ρ*_*e*_, emitted photons 〈*N*〉 and the Mandel’s *Q* parameter as functions of time *t* close to the 2nd resonance for the short and long time scale.

As shown in Fig. 1, the overall dynamic oscillations are presented, especially in the short time scale. Besides, the fast driving-induced oscillation in population *ρ*_*e*_ is observed in the exact results, while it has been averaged by the effective Hamiltonian of GVV and RWA approximations. However, for the photon counting statistics of emitted photons 〈*N*〉 and the Mandel’s *Q* parameter, the fast driving-induced oscillation disappears. As shown in the figure, in this case, the GVV approximation is a good approximation but RWA approximation.

##### *δ*_*off*_ amendment around CDT

**Figure 2:**
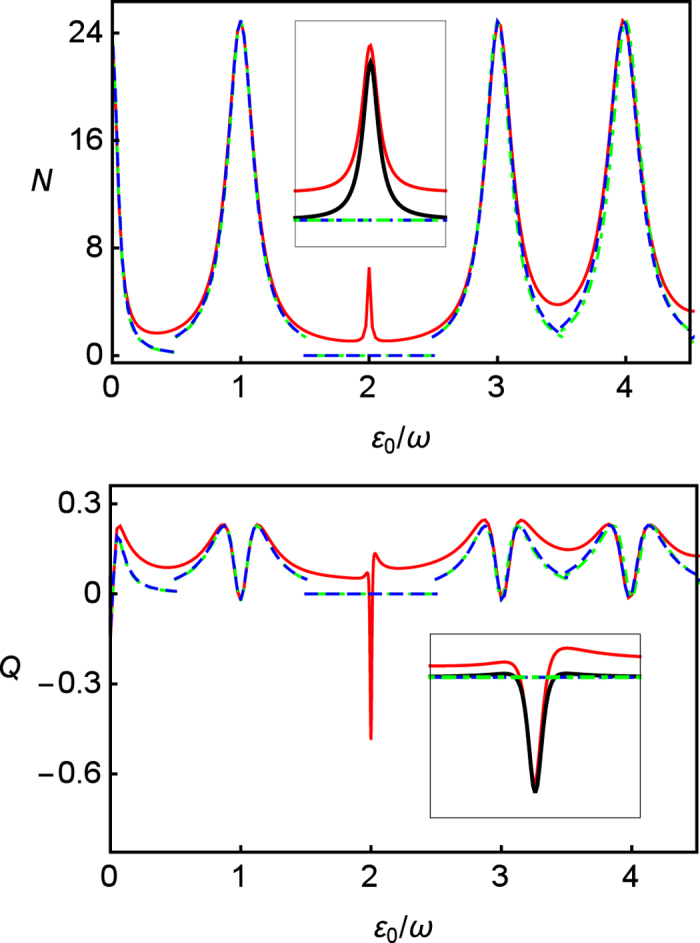
Photon emission 〈*N*〉 (upper) and the Mandel’s *Q* parameter (lower) in long time limit (*t* = 200) as functions of *ε*_0_/*ω*. The parameters are *ω* = 2 (moderate driving), *A*/*ω* = 5.1356 (node point of Bessel function *J*_2_(*z*)), Γ = 0.04, *k*_*p*_ = 0, respectively. The red lines represent exact numerical results, green dash-dotted lines are RWA results, blue dashed lines are the results of the second-order GVV approximation and black lines are the results of the third-order GVV approximation. The insets show details about numerical results (red lines) and the results of the third-order GVV approximation (black lines) around the node point of Bessel function *J*_2_(*A*/*ω* = 5.1356) against *ε*_0_/*ω*. Because the approximations have the discrete effective Hamiltonian in each k-th resonance vicinity, the edges of curves at each resonance are discontinuous^20^.

The contributions of *δ*_*off*_ amendment around CDT are shown in Fig. 2 in the long time limits (*t* = 200). One can find the complete resonance peaks at *ε*_0_/*ω* = 0, 1, 3, 4. At these resonance peaks, the Mandel’s *Q* parameter is less than zero, showing sub-Poissonian distribution. And at off-resonance position, the emitted photons are super-Poissonian distribution since the Mandel’s parameter *Q* > 0. Besides, at the 2nd resonance position (*ε*_0_/*ω* = 2), the results of *Q* under RWA and the second-order GVV approximations are zero, showing a complete CDT occurs. Unfortunately, this is a wrong result, which ignores the CDT shift effect in the weak and moderate driving. By contrast, the exact results show that it is an incomplete CDT phenomenon. The big negative value of the Mandel’s *Q* parameter and its asymmetry (see the following discussion) could be noted as the characteristics of the incomplete CDT phenomenon. As a comparison, the results of the third-order of GVV approximation are also presented in the figure. As shown in the insets of Fig. 2, although the results of third-order of GVV approximation can present the roughly right status, they are different from the exact results.

##### Beyond the third amendment

The results of RWA and GVV approximations present the symmetric feature in the spectra of emitted photons 〈*N*〉 and the Mandel’s *Q* parameter, since the Hamiltonian is symmetric in any *k*-th resonance vicinities under these approximations. However, this symmetric feature is not true except at 0th resonance position (*ε*_0_ = 0). As shown in the inset of Fig. 2 (lower), the exact results indicate the asymmetric feature around the 2nd resonance position, where the 2nd resonance is too weak and dynamical behaviors are modified by nearby resonance peaks in deed. As shown in the figure, the third-order GVV approximation also cannot present this asymmetric feature.

Physically, this asymmetric feature shown in *Q* reflects the intrinsic property of quasi-energy spectrum. In Floquet space, the quasi-energy spectrum against *ε*_0_/*ω* is symmetric around 0th resonance position globally. However, for the *k*-th resonance, this quasi-energy spectrum is local symmetry. These local symmetries are protected by large tunneling Δ_*k*_ around resonance position. However, CDT can break this local symmetries, which is presented on the spectrum of the Mandel’s *Q* parameter significantly because of its sensitivity to the system dynamics.

#### Fast driving 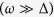


##### The effect of CDT

**Figure 3:**
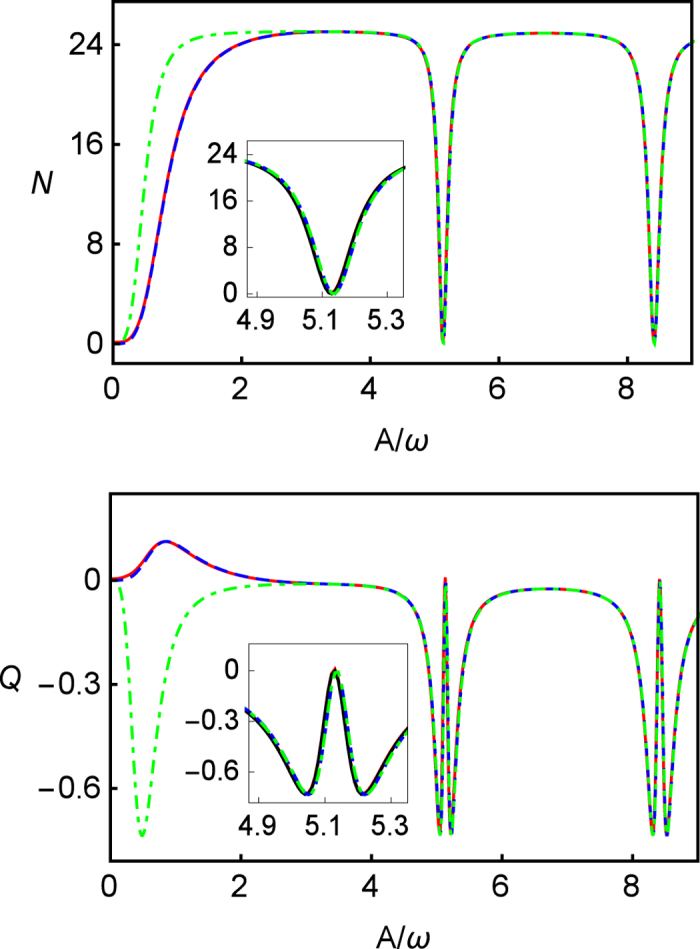
Photon emission 〈*N*〉 (upper) and the Mandel’s *Q* parameter (lower) in long time limit (*t* = 200) as functions of *A*/*ω* at the 2nd resonance. The red lines represent exact numerical results, the green dash-dotted lines are the results of RWA approximation, the blue dashed lines are the results of the second-order GVV approximation, and the black lines are the results of the third-order GVV approximation. The insets show details around *J*_2_(*A*/*ω* = 5.1356) against *A*/*ω* in which the red lines have been covered by black lines. The parameters are *ω* = 5 (fast driving), *ε*_0_/*ω* = 2, Γ = 0.04, *k*_*p*_ = 0.

In Fig. 3, we present the effect of CDT at the 2nd resonance position against *A*/*ω* in fast regime 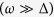. In the weak driving regime (*A* ~ *ω*), the results of RWA deviate with the results of other approaches: the RWA predicts the bigger values of photon emission 〈*N*〉 than others, and shows strong sub-Poissonian distribution. In contrast, the exact numerical results and the results of the second-order GVV approximation indicate super-Poissonian distribution. The reason is, at weak driving regime, that the resonance position is shifted by *δ*_*dia*_.

However, because of fast driving, the results of RWA and the second-order GVV approximation agree with the results of exact numerical calculations at larger driving amplitude. Moreover, *δ*_*off*_ becomes trivial in the node point where CDT occurs. The details around the node point are illustrated in the insets. In Fig. 3, the splitting behaviors of the Mandel’s *Q* parameter are observed. This is because along *A*/*ω*, the resonance oscillation frequency Δ_*R*_ passes through the value of 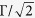 twice, which leads to minimum value of *Q* = −3/4.

**Figure 4:**
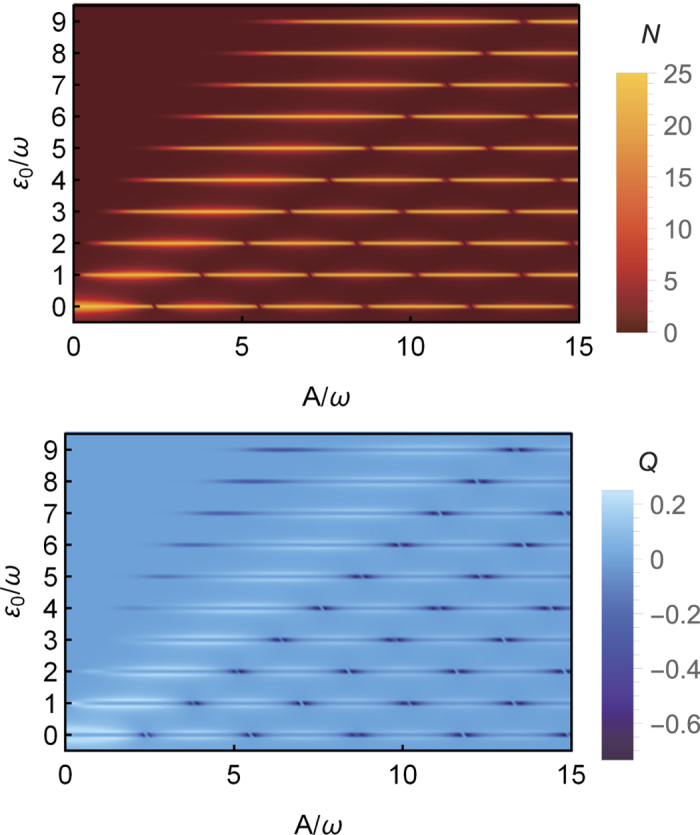
The patterns of photon emission 〈*N*〉 (upper) and the Mandel’s *Q* parameter (lower) in long time limit (*t* = 200) along *A*/*ω* and *ε*_0_/*ω* in fast driving regime (exact numerical results). The parameters are *ω* = 5, Γ = 0.04, *k*_*p*_ = 0.

We illustrate the photon emission pattern along *A*/*ω* and *ε*_0_/*ω* in Fig. 4 based on the exact numerical calculations. The top panel in the figure shows the emitted photons 〈*N*〉, presenting the same pattern with the LZS interference pattern. Meanwhile, the bottom panel in figure shows the Mandel’s *Q* parameter correspondingly. Figure 4 shows, in this weak relaxation regime, a sequence peaks of photon emission and the switches between sub-Poissonian distribution and super-Poissonian distribution against *ε*_0_/*ω* due to *k*-th resonance in TLS. Also, the CDT phenomenon is presented, in the figure, via the photon emitting forbiddenness and splitting behavior of the Mandel’s *Q* parameter against *A*/*ω* at *k*-th resonance peaks.

The results of Fig. 4 clearly show us a widely controlling parameter space to switch off the photon emission process without turning off driving laser field by utilizing the CDT phenomenon. At the same time, this indicates that the maximal sub-Poissonian photon distribution can be obtained by setting appropriate *A*/*ω* around the CDT vicinity.

##### Waiting time in CDT position

Due to the suppression effect of CDT, the waiting time of photon emission becomes much longer, preventing TLS from undergoing spontaneous decay. This allows us to manipulate quantum system with more resilience against decoherence. In Table 1, we show the average waiting time of exact numerical results at CDT position in fast and moderate driving regime. One can find that the fast and strong driving regime will result in longer waiting time. Also we can notice the difference of CDT position between the fast and moderate driving regime due to the contributions of *δ*_*off*_.

### The destruction of CDT

As discussed above, the CDT phenomenon is sensitive to driving condition and only appears in a narrow driving parameter space. In this subsection, we show the CDT is also sensitive to its environment and can be destroyed by strong dissipative effect.

In strong dissipative regime, the TLS is affected by the environment strongly. According to Van Vleck perturbation theory, both inner-system and environment-system interactions are necessary to be considered^34^. However, in GVV approximation, only inner-system interaction is involved^20^,^21^. This indicates, in the case of strong dissipative regime, that the RWA and GVV approaches based on Eqs 8 and 9 do not apply. Since the dynamics of system is dominated by the periodical driving and dissipative rate together, it is convenient to measure dissipative rate in the order of driving frequency *ω*.

In the following, we employ the spectra of emitted photons and the Mandel’s *Q* parameter to present the effects of CDT under the case of strong dissipative regime.

#### CDT destruction by strong relaxation

In this subsection, we study the CDT destruction at the 0th resonance, and consider the TLS under the strong relaxation rate (Γ > *ω*) and fast driving regime 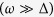.

**Figure 5:**
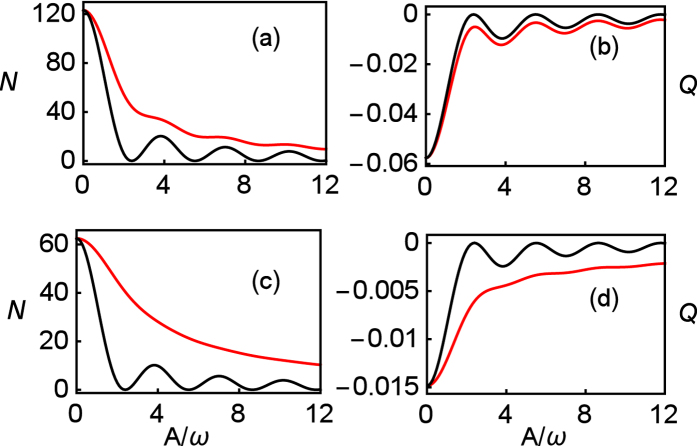
Photon emission 〈*N*〉 and the Mandel’s *Q* parameter in long time limit (*t* = 200) as a function of *A*/*ω*. Panels of (**a**,**b**) are the results for Γ/*ω* = 2 and panels of (**c**,**d**) are the results for Γ/*ω* = 4. The other parameters are *ω* = 5, *ε*_0_ = 0, *k*_*p*_ = 0. The exact numerical results are plotted using red line, and the results of third-order GVV approximation are plotted by black line. Because the results of RWA and second-order GVV approximations overlap with third-order GVV approximation almost, they are not shown.

In strong relaxation regime, the number 〈*N*〉 of emitted photons from TLS is big, and the splitting behaviors of the Mandel’s *Q* parameter would disappear. As expected, the differences between exact numerical results and RWA and GVV results emerge in the panels (a) and (b) of Fig. 5. Further, when Γ is large enough, as shown in the panels of (c) and (d) of Fig. 5, the exact results show the photon emission patterns are monotonous with the increase of driving amplitude parameter *A*/*ω*. In this case, CDT is destroyed by strong relaxation. This differs from the RWA and GVV results significantly.

**Figure 6:**
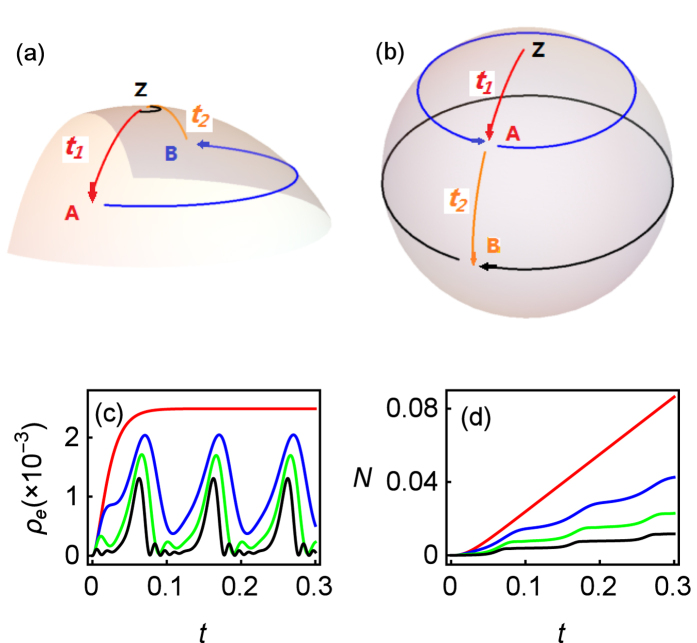
(**a**) Illustration of LZ transition model for CDT phenomenon. Quantum state |*g*〉 in Z point rotates along longitude (red curve) to point A at time *t*_1_ and rotates around *Z* axis along blue curve to point B. Then, at time *t*_2_, quantum state comes back to |*g*〉 in Z point and stays there during another *Z* rotation (black curve). (**b**) Illustration of LZ transition model for *Z*(2*nπ*) case. Quantum state reaches to point B in Bloch sphere after a full driving period. (**c**) *ρ*_*e*_ and (**d**) photon emission 〈*N*〉 of numerical results as a function of dimensionless time *t* for *ω* = 5, *ε*_0_ = 0, *k*_*p*_ = 0, and Γ/*ω* = 4. From upper to lower, each line represents amplitude parameter *A*/*ω* as 0 (red), 4 (blue), 8 (green) and 16 (Black). One can find the up and down of *ρ*_*e*_ and the increasing ladders of *N* induced by diagonal driving.

To understand the novel phenomena presented in Fig. 5, the transfer-matrix approach^31^ based on consecutive LZ transition picture illuminates this situation. In the fast-passage regime 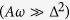, it is convenient to use bases |*g*〉 and |*e*〉 for LZ transition calculation^19^. The consecutive LZ processes form an analog to Mach-Zehnder interferometry^1^. At the time *t*_1_ and *t*_2_ when passing through the avoided crossing (namely, *ε*(*t*_1_) = 0 and *ε*(*t*_2_) = 0), quantum state splits and then accumulates phase in each path consequently. In the case of resonance position, the quantum state will rotate around *X*-axis according to LZ transition probability (*P*_*LZ*_) at *t*_1,2_ and rotate around *Z*-axis according to accumulated phase between *t*_1,2_. For CDT, as shown in panel (a) of Fig. 6, the *Z*-axis rotation is (2*n* + 1)*π*, so in each periodical driving, assuming initial state is |*g*〉, the final state is^19^
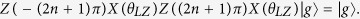


In contrast, as shown in panel (b) of Fig. 6, if the *Z*-rotation is 2*nπ*, the final state will be
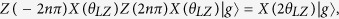
the completed state reverse can occur after several driving period with rotating frequency Δ_*R*_ = |Δ^*g*^|. While for the presence of Γ > *ω*, after each LZ transition, the quantum state will collapse to the ground state |*g*〉 rapidly, and at that point of Bloch sphere, *Z* rotation fails. Thus, in each period *T* = 2*π*/*ω*, the population of excited state *ρ*_*e*_ raises up and drops down twice.

The panel (c) of Fig. 6 shows populations *ρ*_*e*_ as the function of evolution time *t* for the parameters of Γ/*ω* = 4, *ω* = 5, *ε*_0_ = 0. Violent shakes are observed for each line with *A*/*ω* = 4, 8, 16, where the average population of |*e*〉 increases due to LZ transition in *t*_1,2_ and decays subsequently due to strong Γ. While, the upper line is monotonous due to no LZ transition driving, reducing to conventional optical resonance process. Furthermore, according to LZ formalism, the transition probability of |*g*〉 to |*e*〉 can be expressed as
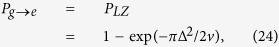
where *v* is cross velocity. And at crossing time *t*_1,2_, the crossing velocity can be expressed by^19^
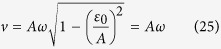


Hence, *P*_*g*→*e*_ is a decreasing function of *A*. However, because of the strong effect of Γ, the average population *ρ*_*e*_ against *A*/*ω* shows the negative correlation property only, rather than strictly follows the formulas of Eqs 24 and 25. Based on this tendency of *ρ*_*e*_, the photon emission from *ρ*_*e*_ will show monotonous property as well.

Considering the population of excited state *ρ*_*e*_ is quite small, we plot the photon emission 〈*N*〉 in the panel (d) of Fig. 6, correspondingly. That shows LZ transition ladders induced by driving. Hence, in this strong relaxation regime, the driving behavior can be observed by photon emission, which differs from weak relaxation regime. In addition, the panel (d) of Fig. 6 also shows that stronger driving would suppress photon emission even though CDT has been destroyed.

#### CDT destruction by strong dephasing

In this subsection, we consider the effect of pure dephasing rate *k*_*p*_ at the 0th resonance under 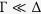 and fast driving regime. If the dephasing rate *k*_*p*_ is much larger than relaxation, the CDT phenomenon could be destroyed by the dephasing rate *k*_*p*_ and shows a different feature from relaxation effect.

**Figure 7:**
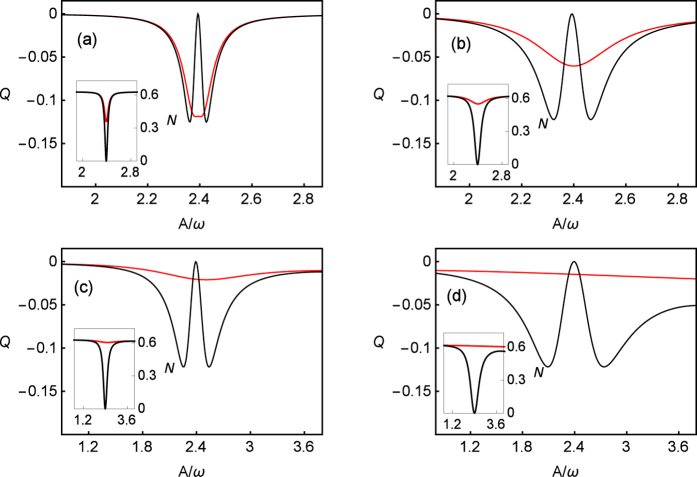
The Mandel’s *Q* parameter (insets: the photon emission 〈*N*〉) in long time limit (*t* = 200) against *A*/*ω* for *k*_*p*_/*ω* = 0.02 (**a**), 0.1 (**b**), 0.4 (**c**) and 2 (**d**) with fixed parameters of *ω* = 5, *ε*_0_ = 0, Γ = 0.001. The exact numerical results are plotted using red lines, and the results of the third-order GVV approximation are plotted using black lines. As *k*_*p*_ increases, even though the results of third-order GVV method broad gradually, the CDT behavior still exists which deviates from correct status.

Figure 7 shows the spectra of the Mandel’s *Q* parameter against *A*/*ω* with the parameters of *ω* = 5, *ε*_0_ = 0, Γ = 0.001 and the spectra of the emitted photons 〈*N*〉 in its insets. Based on the exact numerical calculations, one can observe that the big value of *k*_*p*_ results in the disappearance of CDT at node point of *J*_0_(*A*/*ω*). No matter the spectra of emitted photons 〈*N*〉 or the Mandel’s *Q* parameter, they all become flat upon *A*/*ω*. Along with the decrease of *k*_*p*_, and at node point, the spectra of emitted photons 〈*N*〉 decreases from maximum to 0 gradually. Particularly, the spectra of the Mandel’s *Q* parameter recover the splitting behavior again. However, the results of GVV approximation always demonstrate CDT phenomenon.

In fact, strong dephasing destroys the interference pattern and all frequencies are involved in the node point. Also, the transfer-matrix approach provides strong support for this case. At each crossing time *t*_1,2_, the Bloch vector rotates around *X*-axis by *θ*_*LZ*_. While, due to large *k*_*p*_, the vector transversely collapses to *Z*-axis during the interval of *t*_1,2_. After several similar steps, the vector collapses to the maximal mixed state and this process will not be affected by *A* and *ω* except the rotating angle *θ*_*LZ*_. Hence, as shown in Fig. 7, the spectra of emitted photons 〈*N*〉 and the Mandel’s *Q* parameter are homogeneous against *A*/*ω* and CDT disappears in strong dephasing regime. In addition, due to the influence of small Γ, the flatten lines only slope slightly.

## Conclusion

In this paper, we studied the CDT nature for TLS driven across avoided crossing by employing the photon counting statistics.

In weak relaxation regime, we showed the photon emission spectra calculated by exact numerical, RWA and GVV approximation methods, tested the validity of the approximation methods and explored the new physics beyond them. The asymmetric feature in the spectrum of Mandel’s *Q* parameters nearby CDT position releases an intrinsic asymmetric dynamics of TLS in moderate driving. Also, it is shown that the faster and stronger driving can suppress photon emission and prolong waiting time noticeably via CDT effect.

For the strong dissipative regime, the approximation methods do not apply. The exact numerical results are shown that the CDT is destroyed by strong dissipation. The emitted photons show the monotonicity or homogeneity against *A*/*ω* at resonance position in strong relaxation or dephasing regime.

## Additional Information

**How to cite this article**: Miao, Q. and Zheng, Y. Coherent destruction of tunneling in two-level system driven across avoided crossing via photon statistics. *Sci. Rep.* **6**, 28959; doi: 10.1038/srep28959 (2016).

## Figures and Tables

**Table 1:** Waiting time 〈*τ*〉 in several CDT positions in fast (*ω* = 5) and moderate driving (*ω* = 2) regime at the 2nd resonance position.

**CDT order**	***ω***** = 5**		***ω***** = 2**	
	**〈*****τ*****〉**	***A*****/*****ω***	**〈*****τ*****〉**	***A*****/*****ω***
1	1288.9	5.1280	204.32	5.0864
2	2125.8	8.4122	341.37	8.3854
3	2856.6	11.617	448.63	11.599
4	3588.3	14.794	480.34	14.784
5	4390.1	17.958	573.17	17.951

Other parameters are Γ = 0.04, *k*_*p*_ = 0.
